# “I Don’t Wear Men’s Cloths, I Wear My Own”: How Mary Edwards Walker Pioneered Trauma Surgery as the First Female Trauma Surgeon

**DOI:** 10.1002/wjs.70085

**Published:** 2025-10-03

**Authors:** SaeRam Oh, Alvin Singh, Caitlin A. Fitzgerald, Jill R. Streams

**Affiliations:** ^1^ Department of Surgery Brody School of Medicine at East Carolina University Greenville North Carolina USA; ^2^ Brody School of Medicine at East Carolina University Greenville North Carolina USA; ^3^ Division of Trauma and Acute Care Surgery Brody School of Medicine at East Carolina University Greenville North Carolina USA; ^4^ Division of Acute Care Surgery Vanderbilt University Medical Center Nashville Tennessee USA

**Keywords:** medal of honor, miliary, pioneer in trauma surgery, trauma surgeon, women in surgery

As one of the first female surgeons in the United States, Mary Edwards Walker (1832–1919) stood out by wearing trousers and challenging preexisting gender norms to set a precedent for future female surgeons [1]. Abolitionist, woman's right's activist, and suffragist, Walker was the second woman to have graduated medical school in United States history and served as a surgeon to the 52nd Ohio Volunteers during the Civil War in 1864 [2]. Since 1861, there have been more than 3500 individuals who have received the Congressional Medal of Honor and only one of them is a woman: Dr. Mary Edwards Walker [2]. Initially barred from enlisting in the Union army because she was a woman, Walker volunteered in military hospitals, including one set up in the U.S. Patent Office in Washington, D.C. and was awarded the medal for her service here. However, the medal was revoked in 1917 along with 900 other male recipients for not having been in actual combat after a Congressional review [2]. Notably, this medal was reinstated posthumously in 1977 by President Carter. When asked to return the medal initially, Walker was reported as saying “You may have it back over my dead body and then only if you can get it back from rigor mortis” [2]. After the Civil war had ended, she continued her pursuit for social justice and wrote 2 books fighting for equality and the rights of women. She devoted the rest of her life toward suffrage, dress reform, and public health, leading lectures and writing pamphlets to spread her message [3].

As one of the most pioneering figures in the United States history with innumerable accomplishments and well‐documented contributions, Walker is still underrecognized and often overlooked as the first female trauma surgeon. She played a significant role in the development of the specialty of trauma surgery through her innovative and novel methods. This essay explores her foundational contributions to the field, extending beyond her service as a Civil War surgeon. Her emphasis on immediate intervention (field triage), improved surgical hygiene/antiseptic methods, and unique limb salvage techniques greatly influenced and foreshadowed many principles that would later become cornerstones of modern trauma surgery.

Walker was a leading advocate for immediate medical attention and rapid treatment for wounded soldiers in the Civil War, often treating them herself near combat zones [4, 5]. She was also known to have tended to the medical needs of confederate soldiers as well as nearby civilians, demonstrating her capacity for compassionate care and humane practices. Unfortunately, this led to her capture in 1864 and subsequent release as part of a prisoner of war exchange later that year [5, 6]. Her advocacy for the on‐field stabilization and transportation of injured soldiers laid the groundwork for modern triage and emergency care systems [7]. She often carried the “Pocket Surgical Kit” which carried tools surgeons consider the most useful for emergent care and procedures in the field or austere environment [5]. The current mobile medical hospitals employed by the military medical system mirror Walker's combat treatment practices. Walker also insisted on systematically treating patients with a methodical stepwise approach, a technique that parallels the current advanced trauma life support (ATLS) course and overall trauma systems [8]. Her experience treating severe injuries in high‐stress environments proved invaluable in refining surgical techniques and postoperative care, particularly in preventing infection and complications in trauma patients.

Notably, Walker was a forward‐thinking pioneer advocating for improved surgical hygiene and antiseptic techniques during times when infection was a leading cause of death among soldiers [8]. This predated the widespread acceptance of germ theory as many surgeons were operating with bloody aprons, unwashed hands, and reusing instruments between soldiers. Walker emphasized the need for sterilization of instruments with boiled water, proper wound care with clean bandages, hand and wound washing with running water, and maintaining clean surgical environments [8]. Her emphasis on cleanliness led nurses to bathe patients and change their dressings daily, practices which had not been in place prior to her involvement [8]. Furthermore, during her time of service, she wore a modified Army uniform consisting of trousers, a jacket, and boots. Her change from normal female dress emphasized the unhygienic and nonergonomic nature of traditional women’s clothing in the medical setting [6]. Through these practices, she helped reduce mortality rates of wounded soldiers and prompted further investigation leading to modern antiseptic techniques.

Walker also triumphed in her pioneering work in limb salvage techniques. She advocated for preserving injured limbs rather than early amputation, the primary method of treatment at the time with the estimated rate of mortality of 60% for below the knee amputations and 80% for above the knee amputations [3, 8]. She was known to counsel patients in their rights to refuse amputations and saying, “Many a man today has perfect and good use of his limb who would not have had but for my advice” [3]. She employed advanced wound care techniques, including debridement and irrigation, as well as various procedures including splinting for fracture stabilization and soft tissue coverage to prevent unnecessary amputations [8]. Although performing these early forms of reconstructive surgery, she improved long‐term disability rates as well as psychological outcomes and quality of life for wounded soldiers.

Mary Edwards Walker's influence and legacy as the first female trauma surgeon endures in the field of trauma surgery. Her pioneering work not only shattered gender barriers and inspired early female surgeons but also set the stage for advancements in numerous modern surgical techniques and surgical care in combat medicine. Walker's contributions to the emerging field of trauma surgery have not been well recognized as she did not publish academically. She authored two books during her post‐Civil War career but her clinical career was cut short by illness and injury sustained in the war. In her short time as a surgeon, Dr. Walker successfully changed the landscape of trauma surgery. Her presence endures in the field of trauma surgery as well as among the United States history through items, such as post stamps, a quarter honoring her memory, and the S.S. Mary Walker, a battleship used during WW II in honor of her legacy. Today, her statue in her hometown of Oswego remains as an emblem of her perseverance, innovation, and relentless pursuit of equality in medicine.

## Author Contributions

**SaeRam Oh:** conceptualization, methodology, investigation, resources, writing – original draft, writing – review and editing. **Alvin Singh:** investigation, writing – review and editing. **Caitlin A. Fitzgerald:** investigation, supervision, writing – review and editing. **Jill R. Streams:** conceptualization, methodology, project administration, supervision, writing – review and editing.

## Conflicts of Interest

The authors declare no conflicts of interest.
